# 8p11.23 Amplification in Breast Cancer: Molecular Characteristics, Prognosis and Targeted Therapy

**DOI:** 10.3390/jcm9103079

**Published:** 2020-09-24

**Authors:** Ioannis A. Voutsadakis

**Affiliations:** 1 Algoma District Cancer Program, Sault Area Hospital, 750 Great Northern Road, Sault Ste Marie, ON P6B 0A8, Canada; ivoutsadakis@yahoo.com or ivoutsadakis@nosm.ca; 2 Section of Internal Medicine, Division of Clinical Sciences, Northern Ontario School of Medicine, Sudbury, ON P3E 2C6, Canada

**Keywords:** amplification, breast cancer, chromosome 8p11, amplicon, prognosis, driver oncogene

## Abstract

Background: Amplification of the locus 8p11.23 has been observed in cancer and genes of this locus, including ZNF703 (Zinc finger protein 703), NSD3 (Nuclear receptor binding SET domain protein 3) and FGFR1 (Fibroblast growth factor receptor 1), have been put forward as dominant oncogenes conferring pathophysiologic benefit in cancers with amplifications. However, there is no consensus on the importance of each of them or any other genes of the amplicon or even a consensus on which genes are part of the amplicon. Methods: Publicly available data were used to characterize the locus amplified at 8p11.23 and derive information on each of the genes and roles as oncogenes. The frequency of the amplifications in the locus was examined in the cBioportal platform, and expression levels of the amplicon genes in amplified cases were derived from genomic studies reported in the platform. Examination of the influence of mRNA expressions of each gene of the locus for Recurrence-free survival in breast cancer was performed using K-M plotter. Results: The 8p11.23 amplicon is present in higher frequency in squamous cell lung carcinomas, breast cancers and bladder carcinomas and is only rarely observed in other cancers. The most frequently amplified genes within the amplicon vary between different types of cancers. In breast cancer, amplified cases are most commonly of the luminal B type. Amplified genes are not always over-expressed and there is a low correlation of amplification with over-expression in amplicon genes with variation between genes. The presence of the amplicon does not influence the aneuploidy score or the tumor mutation burden of breast cancers. Regarding prognosis, the two genes of the amplicon whose mRNA hyper-expression portends adverse relapse-free survival in breast cancer are EIF4EBP1 (Eukaryotic transcription initiation factor 4E binding protein 1) and LSM1 (LSM1 homolog, mRNA degradation associated). Conclusion: Besides the previously proposed genes to play a role as dominant oncogenes in the 8p11.23 cancer amplified locus, other genes may also be important in breast cancer based on the high correlation of their amplification and mRNA expression and adverse prognosis conferred by over-expression, consistent with an oncogenic role.

## 1. Introduction

Copy number alterations, both gains and losses, are common molecular lesions in cancer and complement mutations and epigenetic changes in the development of neoplasia [1]. Amplifications of a specific chromosomal locus usually containing several genes are observed in specific cancer types but not in others, implying that the molecular genomic environment promotes and favors stabilization of these loci possibly due to the resident oncogene content. One of the best-known cancer amplicons is observed around the *ERBB2* locus, at chromosome 17q12, in a subset of breast cancers which become sensitive to treatments specifically targeting the over-expressed HER2 (Human EGFR family Receptor 2) receptor. Amplifications of 17q12 are focal and rarely these cancers have polysomy of the whole chromosome [2]. In addition, segments surrounding the amplified locus may be amplified independently or lost, a fact well-illustrated by the gene encoding for topoisomerase II at 17q12, which is commonly amplified or lost in *ERBB2*-amplified breast cancers [3]. The centromeric region of chromosome 17 is often co-amplified with *ERBB2*, but even in these cases there is no associated polysomy of the whole chromosome 17 [4,5]. Amplifications of the locus surrounding *ERBB2* is specific for breast cancer and is rarely seen in other cancer types besides a sub-set of gastric adenocarcinomas [6]. Genes in close proximity to *ERBB2* include *GRB7* (Growth Factor Receptor-Bound protein 7), coding for an adapter protein binding to EGFR (Epidermal Growth Factor Receptor) family tyrosine kinase receptors, and *STARD3* (Steroidogenic Acute Regulatory-related lipid transfer Domain containing 3), encoding for a protein involved in lipid trafficking, are most commonly co-amplified with *ERBB2* [7]. A different amplicon based at locus 11q13 contains the gene *CCND1* encoding for cyclin D which is implicated in hormonal therapy resistance in breast cancer and is targeted therapeutically by inhibitors of cyclin-dependent kinases [8]. Other genes such as those encoding for Fas-Associated Death Domain (*FADD*) or for the cytoskeleton scaffold protein cortactin are also oncogenes frequently co-amplified with *CCND1* at 11q13 or independently in some cases [8].

This paper investigates another amplicon at locus 8p11.23, commonly observed in breast cancers, using publicly available genomic data and other freely available resources informing on the amplicon genes and their products and putative clinical significance. Although several possible oncogenes have been implicated as critical in this locus, there is no clear consensus on the dominant gene or genes that are critical for the establishment and fixation of the amplicon in breast cancer or other cancer types [9,10]. The focus of this investigation is the frequency of the amplicon in different primary cancer types, the most common gene content of the amplified region in breast cancers, expression of the genes commonly amplified and possible prognostic implications. In addition, available data are examined to derive information for the most critical oncogenes residing in the amplicon with a focus on breast cancer.

## 2. Methods

The frequency of amplifications in genes at 8p11 was determined in different studies from The Cancer Genome Atlas (TCGA, www.cancer.gov/about-nci/organization/ccg/research/structural-genomics/tcga) with data listed in the cBioCancer Genomics Portal (cBioportal, http://www.cbioportal.org). cBioportal is a site that enables interrogation of genomic data from publicly available studies [11]. cBioportal associates genomic data in studies from TCGA and other sources with patient clinical characteristics and survival outcomes [12,13,14]. Analysis of copy number alterations in TCGA is performed with the GISTIC (Genomic Identification of Significant Targets in Cancer) algorithm, in which a score of 2 or above denotes putative amplification of a gene. Aneuploidy Score (AS) is calculated as the sum of the number of chromosome arms in each sample that have copy number alterations (gains or losses). A chromosome arm is considered copy number altered, gained or lost, if there is a somatic copy number alteration in more than 80% of the length of the arm, as calculated by the ABSOLUTE algorithm from Affymetrix 6.0 SNP arrays [15]. Chromosomal arms with somatic copy number alterations in 20% to 80% of the arm length are not called and chromosomal arms with somatic copy number alterations in less than 20% of the arm length are considered not altered. mRNA expression grids in cBioportal are constructed and normalized using the RSEM algorithm [16]. The z score used in the mRNA expression comparisons denotes the standard deviations that the expression of a gene in a sample differs to the mean expression of the same gene in samples that are copy neutral for that gene. In addition to the TCGA cohort, analysis for breast cancer was performed with publicly available data from the METABRIC cohort [17]. Two genomic studies of metastatic breast cancer patients, the French INSERM study and the metastatic breast cancer project study, included in cBioportal, were evaluated for determination of the frequency of amplifications of the genes at chromosome 8p11.23 or any additional lesions developing in these genes as breast cancers progress [18].

Expression of proteins of interest from chromosome location 8p11.23 in breast cancer was evaluated using data from the Human Protein Atlas (www.proteinatlas.org), a publicly available database of protein expressions in human normal and neoplastic tissues [19]. The database contains a semi-quantitative immunohistochemistry-based evaluation of the expression of proteins of interest.

Promoter sequences of genes at 8p11.23 as listed in the Eukaryotic Promoter Database (EPD- www.epd.epfl.ch) were interrogated for presence of binding sites of the transcription factors ERα, ERβ, E2F1, FOXA1 and GATA3 as listed in the 8th release of JASPAR open-access database of transcription factors binding profiles [20].

The effect of mRNA expression level of each of the amplicon gene on survival of breast cancer patients was examined with data derived from the online publicly available platform Kaplan Meier Plotter (www.kmplot.com) [21]. The cut-off of amplified and non-amplified samples for each gene was set at the higher quartile of amplification, which is the closer cut-off provided by the platform to the percentage of breast cancer cases with the 8p11 amplicon.

Categorical and continuous data were compared with the Fisher’s exact test or the x^2^ test and the *t*-test, respectively. Kaplan–Meier survival curves were compared using the Log Rank test. All statistical comparisons were considered significant if *p* < 0.05, except for the survival analysis according to mRNA expression levels which was considered significant at a *p* < 0.0005 level to account for multiple comparisons.

## 3. Results

**Figure 1:**
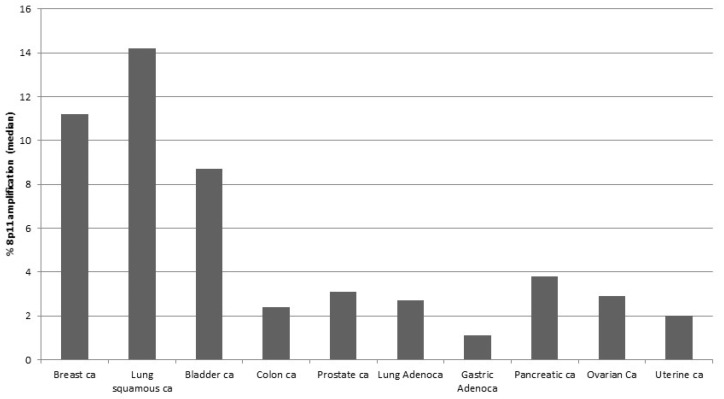
Percentage of the 8p11.23 amplicon expression in common types of cancer. Data are from The Cancer Genome Atlas (TCGA) studies. ca: Cancer.

The previously reported amplified chromosomal area in breast cancer at the short arm of chromosome 8 locus p11 spans an area that contains several coding genes (Table 1). A survey of several TCGA studies shows that the genes at 8p11.23 are amplified as a block in 12% to 17.5% of squamous lung carcinomas, 11% to 12% of breast cancer samples and a slightly lower percentage (7–9%) in bladder cancer samples (Table 2). In several other cancers the amplicon is present in 1% to 4% of cases (Figure 1) and, in these cancers, genes of the locus are co-deleted in a similar percentage of cases (not shown). Co-deletions are also observed in about 3% of bladder cancer cases.

In breast cancer the genes at the most telomeric part of the amplicon, ERLIN2 and ZNF703, display the higher frequency of amplification (11.8% and 11.7% of breast cancer cases, respectively), while the frequency of amplification decreases in more centromeric genes of 8p11.23 locus (Table 3). The most centromeric gene of locus 8p11.23 is FGFR1, which is amplified in 10.9% of breast samples in TCGA. Frequency of amplification further decreases more centromeric to 8p11.23, in genes of loci 8p11.22 and 8p11.21. Those genes are amplified in less than 75% of ZNF703 amplified cases and their expression is not increased in those cases (not shown). The area telomeric to 8p11.23 constitutes a transcribed genes desert and the next genes have a much lower amplification frequency than ERLIN2 and ZNF703. Overall similar but slightly higher, from 13% to 14.3% of the total breast cancer cohort, amplification frequencies are also observed in the METABRIC study, with the most telomeric genes showing again the higher prevalence. In contrast, in squamous carcinomas of the lung, the highest frequency of amplification is observed in the most centromeric genes NSD3, LETM2 and FGFR1 (Table 2).

**Figure 2:**
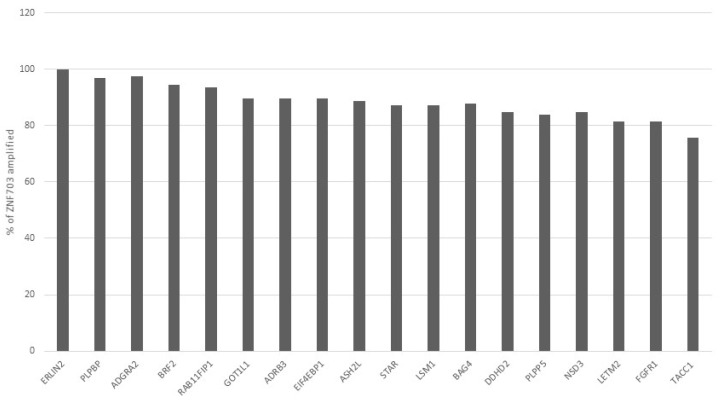
Percentage of cases with amplification of each of the other 8p11.23 genes in samples with ZNF703 amplification in TCGA breast cancer study. The closer neighbors of ZNF703, ERLIN2, ADGRA2, BRF2 and RAB11FIP1 display the higher frequency of co-amplification with a gradual decrease of the frequency of co-amplification in the more remote genes.

The frequency of co-amplification of each of the other genes at 8p11.23 in the 124 samples of breast TCGA study that have ZNF703 amplifications is shown in Figure 2. In samples without ZNF703 amplifications, the rest of the genes of the amplicon are amplified in 0.2% to 1.7% of cases (not shown). Regarding clinicopathologic characteristics, ER+/HER2-/high proliferation cancers were over-represented in the ZNF703-amplified group (as representative of the presence of the whole amplicon) (Table 4). Cancers with positive ER, negative PR and of the luminal B phenotype in the PAM50 classification were also over-represented in the ZNF703-amplified group (Table 4).

**Figure 3:**
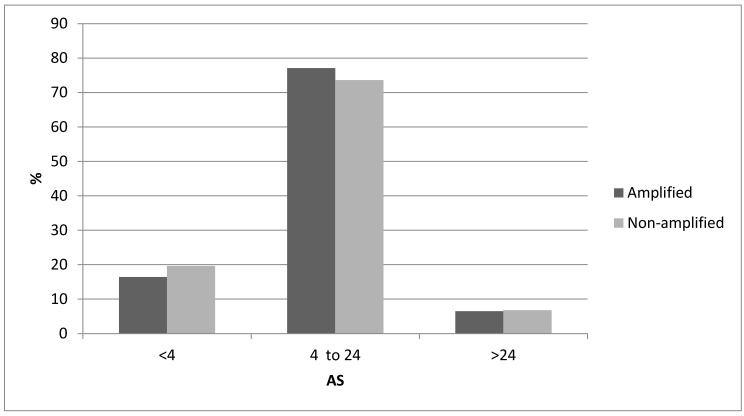
Percentage of cases with the 8p11.23 amplicon and without the amplicon and Aneuploidy Score (AS) of less than 4, between 4 and 24 and above 24. Data from the TCGA study.

**Figure 4:**
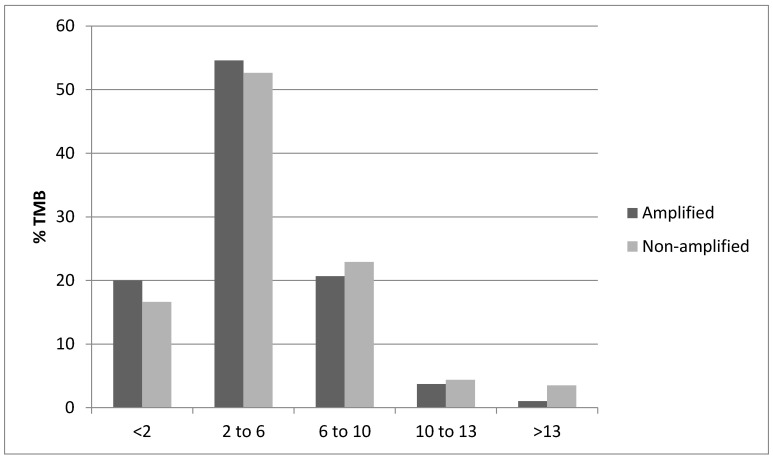
Percentage of cases with the 8p11.23 amplicon and without the amplicon and Tumor Mutation Burden (TMB) of different levels. Data from the METABRIC study.

**Figure 5:**
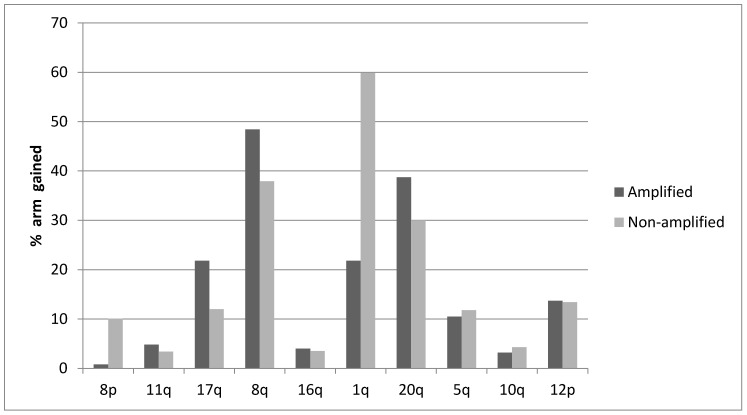
Percentage of chromosome arm gains in breast cancer samples with the 8p11.23 amplicon and without the amplicon in the breast cancer TCGA study.

**Figure 6:**
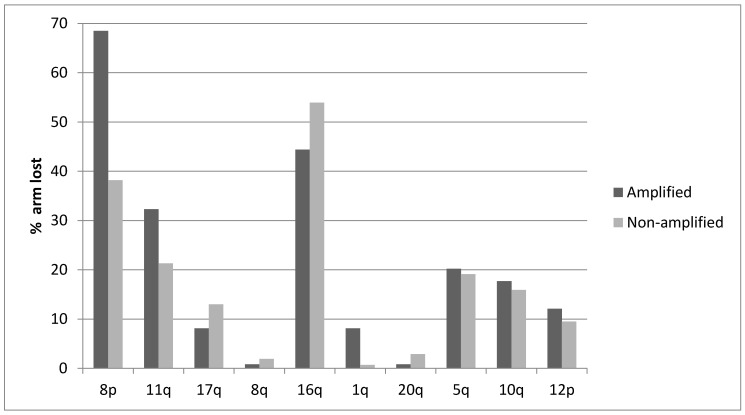
Percentage of chromosome arm losses in breast cancer samples with the 8p11.23 amplicon and without the amplicon.

The distribution of Aneuploidy Scores (ASs) was not significantly different in cancers with ZNF703 amplifications compared with ZNF703 non-amplified breast cancers (Figure 3). The distribution of Tumor Mutation Burden (TMB) was also similar in cancers with ZNF703 amplifications compared with ZNF703 non-amplified cancers, besides the subset with TMB above 13 where non-amplified tumors were more commonly observed, in the METABRIC cohort (Figure 4). However, high TMB frequency is overall low in breast cancer. Frequencies of copy number alterations in specific chromosomal arms differ significantly between ZNF703-amplified and non-amplified cases. Most interestingly, gain of 8p arm is only observed in one case (0.8%) in the ZNF703-amplified cohort and in 10.1% of cases in the non-amplified cohort (x^2^ *p* < 0.001) (Figure 5). In contrast losses of chromosome arm 8p are present in 68.5% of ZNF703-amplified cases and in only 38.2% of ZNF703 non-amplified cases (x^2^ *p* < 0.001) (Figure 6). Other chromosome arms with significant differences in the frequencies of putative copy number alterations between the ZNF703-amplified and non-amplified cohorts in the breast cancer TCGA study include 17q and 1q (x^2^ *p* = 0.006 and 0.004, respectively) (Figure 5).

**Figure 7:**
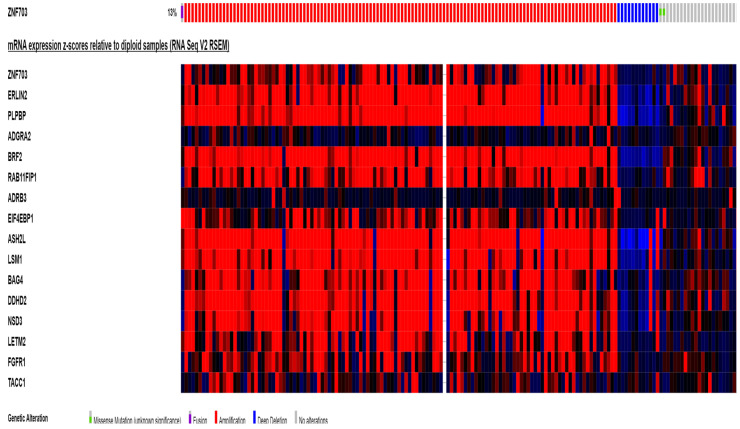
mRNA expression of amplicon genes in cases with ZNF703 amplification (as a marker of amplicon presence). In the right side of the grid, cases with deletion of the amplicon or normal expression are shown for comparison.

Evaluation of the median mRNA expression of genes of the amplicon in TCGA breast cancer study discloses that a few genes (GOT1L1, ADRB3, STAR and LETM2) have a very low or no expression in breast cancers. Among these, ADRB3 and STAR as well as ADGRA2 show no expression at the protein level at Human Protein Atlas and thus are unlikely to be important in breast carcinogenesis. Moreover, these genes, as well as ZNF703, RAB11FIP1, EIF4EBP1 and FGFR1 show low correlation in amplified cases with increased mRNA expression in TCGA (Figure 7). In contrast, ERLIN2, PLPBP, BRF2, ASH2L, BAG4 and NSD3 are most often upregulated at the mRNA level in cases with amplifications of the 8p11.23 amplicon. Among the 150 ER+/HER2-/Proliferation high breast cancers in the ZNF703-amplified group of the METABRIC study cohort, the most commonly putative increased mRNA expression (z score above 2) was observed in BRF2 (46% of cases), ERLIN2 (42.7% of case), NSD3 (40.7% of cases), ASH2L (39.3% of cases), RAB11FIP1 (38.7% of cases) and PLPBP (38.7% of cases). Other genes of the amplicon show lower over-expression frequencies in ER+/HER2-/Proliferation high breast cancers (Table 5).

A survey of promoter binding sequences disclosed that all genes in the amplicon possess several putative binding sequences in their promoters for ERα, ERβ and the transcription factor E2F1 which is a target of activation by the cyclin D/CDK4 cascade in breast cancer with high proliferation fraction (Table 6). In addition, several but not all genes possess promoter binding sites for the breast cancer pioneer factors FOXA1 and GATA3 and the transcription factor NFE2L2, a master regulator of detoxification programs which has been proposed to co-operate with BRF2 in resetting the cell oxidative stress tolerance limit (Table 6) [22].

Expression of the protein products of 8p11.23 genes in breast cancer, using immunohistochemistry with commercially available monoclonal and polyclonal antibodies, is shown in Table 7. This evaluation includes cases with and without 8p11.23 amplifications. Expression of the proteins in breast cancers independently of sub-type and amplicon presence implies a potential of the protein to be a pathogenic player. Most proteins are moderately to highly expressed in several breast cancer cases with at least one antibody checked, the exception being ADGRA2, ADRB3 and STAR, whose genes are also not over-expressed at the mRNA level.

Next, the evolution of the 8p11.23 amplifications in metastatic breast cancer studies was assessed. In two studies, that included metastatic breast cancer patients, a slight increase in the frequency of amplifications was observed compared with non-metastatic studies (Table 8). In one of the studies, the metastatic breast cancer project study, with 180 metastatic breast cancer patients, ZNF703 amplifications were more significantly increased and were observed in 20.6% of patients, including six samples with isolated amplifications of the gene without the neighboring genes being amplified. However, no concomitant increased ZNF703 mRNA expression was observed in these samples. In addition, mutations in any of the amplicon genes remain rare in metastatic breast cancer with a frequency of 1.4% or lower (range of mutated samples in each amplicon gene in either study = 0 to 4). In the French INSERM study, ZNF703, ERLIN2 and PLPBP were the most frequently amplified genes in 34 of 216 (15.7%) of patients [18].

Another commonly amplified chromosomal locus in breast cancer is found at 11q13 and has been reported to be commonly co-amplified with 8p11 amplicon [23]. Interrogation of TCGA breast cancer cohort confirmed that the 11q13 amplification (as captured by amplification of CCND1 gene) is observed in 34.7% of cases with the 8p11.23 amplicon (as represented by amplification of ZNF703 gene). In contrast, the 11q13 amplification is observed in only 12.5% of cases without 8p11.23 amplifications (x^2^ *p* < 0.0001). Among the 43 cases in TCGA with 8p11 and 11q13 co-amplifications, 51.2% were of the luminal B subtype and 41.9% were of the luminal A subtype. These sub-types represent about 15% and 50%, respectively, of the total number of cases in the TCGA breast cancer study. The length of 8p11.23 amplicon, as determined by the number of amplified genes, is not different when 11q is co-amplified, compared with samples that do not possess 11q13 amplification.

**Figure 8:**
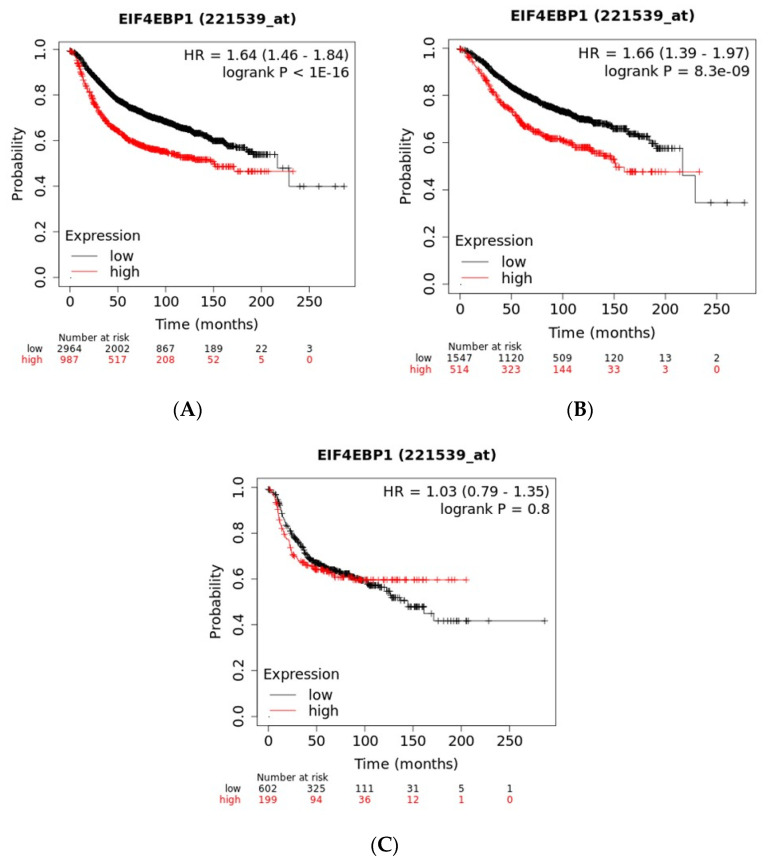
Recurrence-free survival in patients with high mRNA expression of EIF4EBP1 (the higher quartile of the cohort) compared with patients with lower mRNA expression. (**A**) Across breast cancer sub-types; (**B**) in ER (Estrogen Receptor) positive breast cancers; (**C**) in ER negative breast cancers.

**Figure 9:**
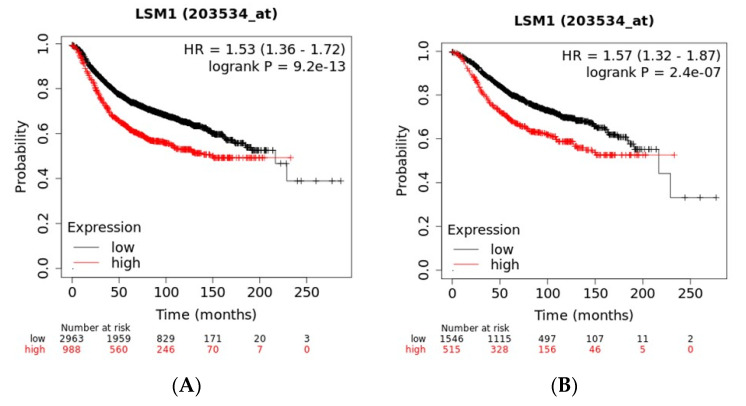

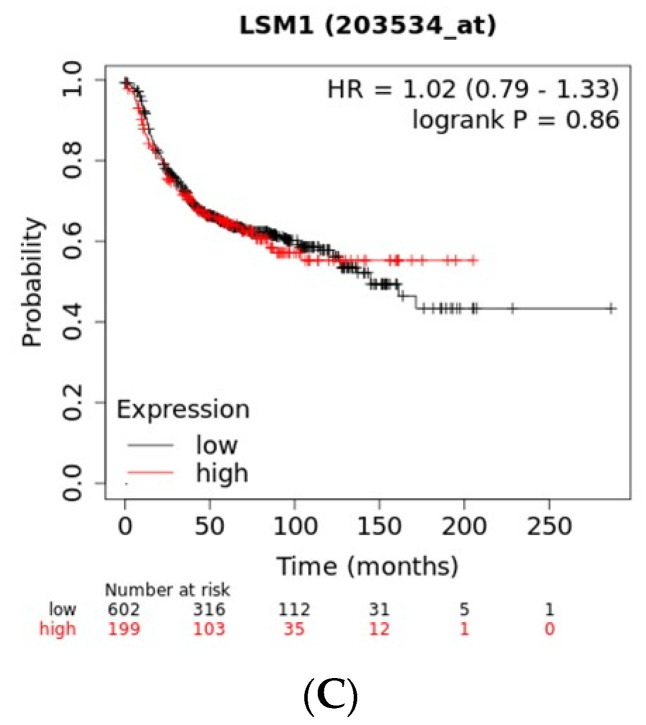
Recurrence-free survival in patients with high mRNA expression of LSM1 (the higher quartile of the cohort) compared with patients with lower mRNA expression. (**A**) Across breast cancer sub-types; (**B**) in ER positive breast cancers; (**C**) in ER negative breast cancers.

**Figure 10:**
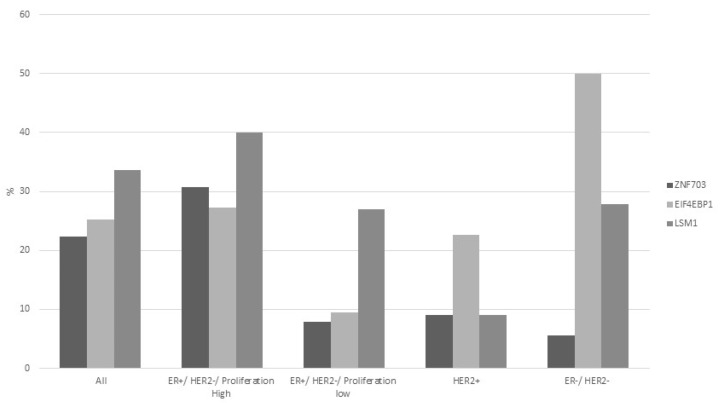
Increased mRNA expression of EIF4EBP1, LSM1 and ZNF703 in samples with amplification of 8p11.23 according to the 3-gene classifier. Percentage of cases with a z score above 2 for each of the 3 genes is shown.

Prognosis of breast cancer patients with the higher quartile mRNA expressions of each of the amplicon genes was compared with counterparts with mRNA expressions at the three lower quartiles. Among cohorts of patients with all sub-types of breast cancer, patients with higher expression (the higher quartile of the cohorts) of EIF4EBP1 and LSM1 mRNA had worse Relapse-Free Survival (RFS) than patients with lower expression of either genes, suggesting that the two genes act as oncogenes (Figure 8A and Figure 9A). When examined according to breast cancer sub-type, worse RFS of high expressers was observed in ER (Estrogen Receptor) positive cancers (Figure 8B and Figure 9B) but not in ER negative cases (Figure 8C and Figure 9C). The frequency of mRNA over-expression (z score > 2) in the different sub-groups of luminal breast cancers classified according to the 3-gene classifier for EIF4EBP1 and LSM1 was higher for the ER+/HER2-/proliferation high group compared with the ER+/HER2-/proliferation low group, suggesting that, in both gene cases, increased gene dosage in luminal B cancers translates in higher mRNA production and possibly increased protein that could lead to inferior RFS outcomes (Figure 10).

RFS of cohorts of breast cancer patients categorized according to mRNA expressions of the other genes of the amplicon showed no statistically significant difference between the higher quartile expression and lower quartiles. For ERLIN2, NSD3, LETM2, FGFR1 and TACC1 cases with higher mRNA expression had even a better survival than the cohorts with lower expressions, although not reaching statistical significance at the pre-set 0.0005 level.

## 4. Discussion

Copy number alterations are more common than mutations in breast cancer and often happen in clusters encompassing several genes. One of the most common clusters of amplification is observed at locus 8p11.23 in about 10% to 15% of the total breast cancer cases. Nineteen genes are located on the most frequently amplified segment. In the great majority of amplified cases the amplification encompasses the whole segment with all the genes amplified, and more rarely only parts of the segment, most commonly including the more telomeric area.

The 8p11.23 amplicon or areas close to it at 8p have been previously reported to play an oncogenic role in breast cancer and putative driver oncogenes among the genes located in the amplicon have been proposed. These include ZNF703, FGFR1, EIF4EBP1, LSM1, BAG4 and PLPP5 [24]. ZNF703 amplifications, for example, are associated with PR negativity among ER positive breast cancers [25]. ER positive/PR negative/HER2 negative cancers segregate in the luminal B genomic phenotype, are commonly endocrine therapy resistant and have poor prognosis [26]. A survey of genome gains and losses in luminal and basal breast cancers using array comparative genomic hybridization (aCGH) microarrays disclosed that ZNF703 was the most significant candidate oncogene in luminal cancers [27]. ZNF703 mRNA over-expression correlates better with gene amplification of the gene in luminal B cancers and was associated with worse overall survival [28]. Breast cancer cell lines expressing ZNF703 were resistant to tamoxifen treatment, while down-regulation of ZNF703 mRNA through miRNA synergized with tamoxifen in cell killing [29]. ZNF703 is an ER-responsive gene and has a negative effect in expression of ER by suppressing its promoter in a negative feedback loop [30]. In addition, ZNF703 up-regulates transcription factor E2F1, having, hence, a role in promoting proliferation [30,31]. ZNF703 suppresses also TGFβ signaling in breast cancer cells, neutralizing TGFβ-mediated anti-proliferative transduction signals [32]. These data place ZNG703 as a strong candidate oncogene in breast cancers with amplifications of its gene locus. 

FGFR1 overexpression has been reported to promote endocrine therapy resistance and to decrease DMFS (Distant Metastasis Free Survival) in ER positive cancers. Moreover, it is associated with higher Ki67 and decreased PR expression both characteristics of luminal B subtype [33]. Amplification of EIF4EBP1 may favor the common co-occurrence of 8p11 amplification with amplifications of 11q13 which have been previously reported and are confirmed in the current study. The co-occurrence of 11q13 and 8p11 amplifications leads, among other genes, to amplification of gene RPS6KB2, encoding for kinase S6K2, on 11q13, which is, in common with EIF4EBP1, a target of mTOR (mechanistic Target of Rapamycin) complex and co-operates with it in cell programs of protein synthesis for cell growth [34]. ER positive/HER2 negative breast cancers that were resistant to short term letrozole neo-adjuvant therapy and remained highly proliferative as measured by Ki67 expression were amplified for 8p11 and 11q13 [35]. Cyclin D amplification from 11q13 leads to up-regulation of function of transcription factor E2F1, which then promotes transcription of ZNF703 and FGFR1 genes of the 8p11.23 amplicon [23]. ZNF703 induction completes a positive feedback loop, given that ZNF703 is an inducer of E2F1 [30,36].

The current descriptive investigation based on the published series details the characteristics of 8p11.23 amplicon in breast cancer and elucidates possible implications based on gene expressions and regulations. Amplifications of 8p11.23 are not unique for breast cancer but are observed also in squamous lung carcinomas and urothelial cancers. In contrast, they are more rarely observed in other cancers including lung adenocarcinomas and other non-lung squamous carcinomas. This implies that one or more amplified genes in the area are under positive pressure for over-expression in some cancer environments but not others. This notion is enforced by the fact that in breast cancer there is a higher prevalence of the amplicon in aggressive luminal cancers compared with other sub-types. The gene or genes that are drivers of the aggressive pathophysiology of the amplified cases act through direct signaling effects and not through a global influence on the cancer tumor mutation burden or ploidy status, as there are no significant differences in TMB and AS in 8p11.23 amplified and non-amplified breast cancers. 

A higher probability that the amplification of a gene of the amplicon is a driver event would be expected if the amplification is associated with a higher expression of the gene products at the mRNA and protein levels, and if higher expression is associated with adverse patient prognosis. Among the genes of the amplicon, mRNA expression is imperfectly associated with amplification with the higher number of cases with mRNA over-expression observed for ERLIN2, PLPBP, BRF2, RAB11FIP1, ASH2L, LSM1, DDHD2 and NSD3 in cancers with the amplicon. An adverse prognosis for higher mRNA expression was only present for EIF4EBP1 and LSM1. The caveat of this evaluation is that the comparison was made between groups expressing the respective mRNAs above or below the upper quartile and, as a result, several of the cases included in the high group would be non-amplified for the respective genes. The expression of the protein products of the amplicon genes is observed in several breast cancers; a fact that would maintain them in the list of candidate drivers. This evaluation is not able to inform regarding the functional status of the proteins or the presence and function of different isoforms that exist.

mRNA expression and eventual protein expression of the amplified genes may still depend on the presence of transcription factors and programs that are critical for their transcription in cells without the amplifications. In luminal cancers, the ER-dependent programs are influenced by pioneer factors FOXA1 and GATA3 and thus genes whose promoters contain binding sites or clusters of sites for these factors may be expected to be dependent on ER programs [37,38]. However, in luminal B cancers, where the amplicon is more often present, E2F1 programs, dependent on CDK4/cyclin D activation, may substitute for ER programs, which have lower activity in these cancers [39]. The presence of binding sites for both the ER axis and E2F1 in many promoters of amplicon genes confirms the potential for regulation that could switch from ER dominant in luminal A cancers to E2F1 programs in luminal B cancers, or upon progression, when hormonal resistance develops. In the evaluation of protein products expression of the genes in 8p11.23 most, besides ADGRA2, ADRB3 and STAR are confirmed to be present. Variability in the expression with different monoclonal and polyclonal antibodies used in the Human Protein Atlas is probably a result of both the antigen specificity of the antibody, a variability of expression between cases and possibly the presence of homologous proteins in the human genome that could cross react.

A significant percentage (almost 70%) of 8p11.23 amplified breast cancer cases in TCGA display a global loss of the 8p arm denoting that the 8p11.23 locus is amplified amidst extensive chromosomal material losses elsewhere in the 8p arm. 8p losses are less common (less than 40% of cases) in 8p11.23 non-amplified breast cancers. This suggests a mechanism of acquisition of the amplicon in which it arises in cases with random extensive 8p arm breaks, that lead mostly to chromosomal material loss, and is favored and becomes fixed due to promotion of survival and proliferation in the cancer cells harboring it. It would be worth investigating whether the ratio of amplification compared to loss of surrounding loci suggests the presence of an oncogene more globally and could serve for the establishment of criteria of oncogene discovery.

Regarding therapy of breast cancers harboring the 8p11.23 amplicon, FGFR inhibitors have been investigated and could be a rational targeted therapy for cancers overexpressing FGFR1. However, the clinical experience with FGFR inhibitors shows that cancers with FGFR mutations or fusions are more sensitive to inhibition compared to cancers that harbor amplifications [40,41]. It is possible that, akin to HER2 inhibitors, the level of amplification of FGFR1 and the level of protein expression will need to be taken into consideration in the targeted development of FGFR inhibitors for the therapy of 8p11.23 amplified breast cancers and other types of cancer with the amplicon.

EIF4EBP1 is a negative regulator of protein translation and is a target of kinase mTOR which inhibits EIF4EBP1, thereby promoting protein translation that is critical in proliferating cells [42]. Breast cancer with 8p11.23 inhibition of the amplified EIF4EBP1 may increase the dependency of the cancer cells to the activity of mTOR in order to secure active protein production, and thus may make these cells sensitive to inhibition of mTOR by drugs such as everolimus. Interestingly, a protein produced by a gene in the frequently co-amplified 11q13 amplicon, p70S6 is also an mTOR target in a cell growth pathway, possibly increasing the dependence of co-amplified cells to mTOR inhibitors. Presence of 8p11.23 and/or 11q13 amplicons as predictive biomarkers of mTOR inhibition efficacy remains to be determined in clinical studies. Other therapeutic opportunities relying on putative tumor cell dependencies on products of 8p11.23 amplified genes may exist but would require further studies. For example, inhibition of histone modifiers BRF2 and NSD3 could be a targeted approach of interest, should clinical-grade inhibitors become available for development.

## Figures and Tables

**Table 1:** Genes at the 8p11.23 locus. Positions are according to human genome version GRCh38.

Official Name	Name Spell-Out/Function	Ensembl Number	Alternative Names	Position	Strand
ZNF703	Zinc finger protein 703	ENSG00000183779	FLJ14299, ZEPPO1	37,695,782–37,700,019	Forward
ERLIN2	Endoplasmic reticulum lipid raft associated 2	ENSG00000147475	SPFH2	37,736,601–37,758,422	Forward
PLPBP	Pyridoxal phosphate binding protein	ENSG00000147471	PROSC	37,762,595–37,779,768	Forward
ADGRA2	Adhesion G protein-coupled receptor A2	ENSG00000020181	GPR124	37,784,191–37,844,896	Forward
BRF2	RNA polymerase III transcription initiation factor sub-unit	ENSG00000104221	TFIIIB50	37,843,268–37,849,861	Reverse
RAB11FIP1	RAB11 family interacting protein 1	ENSG00000156675		37858618–37899497	Reverse
GOT1L1	Glutamic-oxaloacetic transaminase 1 like 1	ENSG00000169154	MGC33309	37,934,281–37,940,124	Reverse
ADRB3	Adrenoreceptor beta 3	ENSG000000188778		37,962,990–37,966,599	Reverse
EIF4EBP1	Eukaryotic transcription initiation factor 4E binding protein 1	ENSG00000187840	4E-BP1	38,030,534–38,060,365	Forward
ASH2L	ASH2 like histone lysine methyltransferase complex sub-unit	ENSG00000129691		38,105,493–38,144,076	Forward
STAR	Steroidogenic acute regulatory protein	ENSG00000147465	STARD1	38,142,700–38,150,992	Reverse
LSM1	LSM1 homolog, mRNA degradation associated	ENSG00000175324	CASM	38,163,335–38,176,730	Reverse
BAG4	BAG cochaperone 4	ENSG00000156735	SODD	38,176,533–38,213,301	Forward
DDHD2	DDHD domain containing 2	ENSG00000085788		38,225,218–38,275,558	Forward
PLPP5	Phospholipid phosphatase 5	ENSG00000147535	PPAPDC1B, HTPAP	38,263,130–38,269,243	Reverse
NSD3	Nuclear receptor binding SET domain protein 3	ENSG00000147548	WHSC1L1	38,269,704–38,382,272	Reverse
LETM2	Leucine zipper and EF-hand containing transmembrane protein 2	ENSG00000165046	SLC55A2	38,386,207–38,409,527	Forward
FGFR1	Fibroblast growth factor receptor 1	ENSG00000077782	CD331	38,400,215–38,468,834	Reverse
TACC1	Transforming acidic coiled-coil containing protein 1	ENSG00000147526		38,728,186–38,853,028	Forward

GRCh38: Genome Reference Consortium human genome assembly, version 38.

**Table 2:** Amplifications of 8p11.23 genes in TCGA studies of different cancers. KAT6A from 8p11.21 is included for comparison. In parentheses in the title line the total number of samples in each study is presented. In parentheses in subsequent lines are the percentages of amplifications.

8p11.23 Genes	Breast Cancer (1070)	Bladder Cancer (408)	Lung Squamous Carcinoma (487)	Colon Cancer (592)	Prostate Cancer (489)	Lung Adenocarcinoma (511)	Gastric Adenocarcinoma (438)	Pancreatic Cancer (183)	Ovarian Cancer (572)	Endometrial Cancer (523)
ZNF703	125 (11.7)	39 (9.6)	56 (11.5)	9 (1.5)	16 (3.3)	13 (2.5)	5 (1.1)	6 (3.3)	15 (2.6)	6 (1.1)
ERLIN2	126 (11.8)	40 (9.8)	62 (12.7)	10 (1.7)	15 (3.1)	13 (2.5)	5 (1.1)	7 (3.8)	16 (2.8)	6 (1.1)
PLPBP	122 (11.4)	40 (9.8)	62 (12.7)	9 (1.5)	15 (3.1)	13 (2.5)	5 (1.1)	7 (3.8)	16 (2.8)	6 (1.1)
ADGRA2	125 (11.7)	41 (10)	64 (13.1)	9 (1.5)	15 (3.1)	15 (2.9)	5 (1.1)	7 (3.8)	15 (2.6)	9 (1.7)
BRF2	121 (11.3)	40 (9.8)	63 (12.9)	10 (1.7)	15 (3.1)	15 (2.9)	5 (1.1)	7 (3.8)	15 (2.6)	9 (1.7)
RAB11FIP1	122 (11.4)	40 (9.8)	65 (13.3)	10 (1.7)	15 (3.1)	15 (2.9)	5 (1.1)	7 (3.8)	17 (3)	9 (1.7)
ADRB3	120 (11.2)	35 (8.6)	66 (13.6)	9 (1.5)	15 (3.1)	15 (2.9)	5 (1.1)	7 (3.8)	16 (2.8)	10 (1.9)
EIF4EBP1	120 (11.2)	36 (8.8)	68 (14)	14 (2.4)	15 (3.1)	16 (3.1)	5 (1.1)	7 (3.8)	16 (2.8)	10 (1.9)
ASH2L	120 (11.2)	35 (8.6)	69 (14.2)	16 (2.7)	15 (3.1)	15 (2.9)	5 (1.1)	7 (3.8)	17 (3)	11 (2.1)
LSM1	119 (11.1)	35 (8.6)	69 (14.2)	15 (2.5)	15 (3.1)	14 (2.7)	5 (1.1)	7 (3.8)	17 (3)	11 (2.1)
BAG4	120 (11.2)	35 (8.6)	69 (14.2)	15 (2.5)	15 (3.1)	14 (2.7)	5 (1.1)	7 (3.8)	16 (2.8)	12 (2.3)
DDHD2	117 (10.9)	35 (8.6)	76 (15.6)	16 (2.7)	15 (3.1)	14 (2.7)	6 (1.4)	7 (3.8)	16 (2.8)	12 (2.3)
PLPP5	115 (10.7)	35 (8.6)	76 (15.6)	17 (2.9)	15 (3.1)	14 (2.7)	6 (1.4)	7 (3.8)	16 (2.8)	12 (2.3)
NSD3	120 (11.2)	36 (8.8)	86 (17.7)	27 (4.6)	15 (3.1)	14 (2.7)	6 (1.4)	7 (3.8)	18 (3.1)	12 (2.3)
LETM2	116 (10.8)	32 (7.8)	85 (17.5)	26 (4.4)	15 (3.1)	13 (2.5)	6 (1.4)	7 (3.8)	18 (3.1)	12 (2.3)
FGFR1	117 (10.9)	33 (8.1)	83 (17)	24 (4.1)	15 (3.1)	14 (2.7)	6 (1.4)	7 (3.8)	18 (3.1)	12 (2.3)
TACC1 (8P11.22)	111 (10.4)	32 (7.8)	72 (14.8)	21 (3.5)	14 (2.9)	15 (2.9)	4 (0.9)	7 (3.8)	18 (3.1)	12 (2.3)
KAT6A (8p11.21)	85 (7.9)	25 (6.1)	37 (7.6)	24 (4.1)	10 (2)	20 (3.9)	10 (2.3)	4 (2.2)	27 (4.7)	29 (5.5)

TCGA: The Cancer Genome Atlas, KAT6A: Lysine Acetyl-Transferase 6A.

**Table 3:** Number and percentage of cases with amplifications of the 8p11.23 genes and, for comparison, genes of 8p11.21, in TCGA breast cancer and METABRIC studies.

Genes at 8p11.23	TCGA (%)	METABRIC (%)	Genes at 8p11.21	TCGA (%)	METABRIC (%)
ERLIN2	126 (11.8)	305 (14)	ADAM9	106 (9.9)	244 (11.2)
ZNF703	125 (11.7)	310 (14.3)	ADAM32	104 (9.7)	242 (11.1)
PLPBP	122 (11.4)	303 (13.9)	ADAM5	99 (9.3)	268 (13.1)
ADGRA2	125 (11.7)		ADAM2	98 (9.2)	239 (11)
BRF2	121 (11.3)	297 (13.7)	ZMAT4	96 (9)	228 (10.5)
RAB11FIP1	122 (11.4)	293 (13.5)	SFRP1	84 (7.9)	217 (10)
ADRB3	120 (11.2)	289 (13.3)	SLD5	84 (7.9)	225 (10.4)
EIF4EBP1	120 (11.2)	291 (13.4)	ANK1	85 (7.9)	239 (11)
ASH2L	120 (11.2)	290 (13.3)	KAT6A	85 (7.9)	235 (10.8)
LSM1	119 (11.1)	289 (13.3)	IKBKB	82 (7.7)	223 (10.3)
BAG4	120 (11.2)	292 (13.4)	POLB	80 (7.5)	220 (10.1)
DDHD2	117 (10.9)	293 (13.5)	DKK4	80 (7.5)	217 (10)
NSD3	120 (11.2)	291 (13.4)	VDAC3	80 (7.5)	218 (10)
LETM2	116 (10.8)	289 (13.3)	SLC20A2	82 (7.7)	208 (9.6)
FGFR1	117 (10.9)	285 (13.1)	RNF170	74 (6.9)	196 (9)
TACC1 (8p11.22)	111 (10.4)	265 (12.2)	FNTA	84 (7.9)	191 (8.8)

TCGA: The Cancer Genome Atlas, METABRIC: Molecular Taxonomy of Breast Cancer International Consortium.

**Table 4:** Comparison of ZNF703 amplified (*n* = 310) and non-amplified (*n* = 2199) cases in the METABRIC study.

	Amplified (%)	Non-Amplified (%)	*p*
**Histology**			
IDC	252 (81.3)	1613 (73.4)	0.2
ILC	20 (6.5)	172 (7.8)	
IMixed	26 (8.4)	243 (11.1)	
Other	7 (2.3)	41 (1.8)	
NA	5 (1.6)	130 (5.9)	
**Three-Gene classifier**			
ER+/HER2-/Proliferation High	153 (49.4)	464 (21.1)	<0.000
ER+/HER2-/Proliferation Low	63 (20.3)	577 (26.2)	
HER2+	23 (7.4)	175 (8)	
ER-/HER-	19 (6.1)	290 (13.2)	
NA	52 (16.8)	693 (31.5)	
**ER status**			
positive	262 (84.5)	1555 (70.7)	<0.000
negative	41 (13.2)	568 (25.8)	
NA	7 (2.3)	76 (3.5)	
**PR status**			
positive	128 (41.3)	912 (41.5)	0.02
negative	150 (48.4)	790 (35.9)	
NA	32 (10.3)	497 (22.6)	
**Grade**			
1	19 (6.1)	195 (8.9)	0.02
2	110 (35.5)	866 (39.4)	
3	171 (55.2)	1027 (46.7)	
NA	10 (3.2)	111 (5)	
**PAM50 type**			
Luminal A	84 (27.1)	616 (28)	<0.000
Luminal B	106 (34.2)	369 (16.8)	
HER2	30 (9.7)	194 (8.8)	
Basal-like	17 (5.5)	192 (8.7)	
Claudin-low	20 (6.5)	198 (9)	
Normal-like	20 (6.5)	128 (5.8)	
NA/NC	33 (10.6)	497 (22.6)	

NA: Not Available, IDC: Invasive Ductal Carcinoma, ILC: Invasive Lobular Carcinoma, IMixed: Invasive Mixed Ductal and Lobular Carcinoma. x^2^ test *p* values in the last column refer to comparisons between the amplified and non-amplified groups, with the cases for which there is no available information for the characteristic compared excluded.

**Table 5:** Expression of amplicon genes in amplified samples from METABRIC according to the 3-gene classifier. Data on the classifier are available from 258 of 310 total patients with ZNF703 amplifications. Data on the mRNA z score are available from 273 of 310 total patients. Some patients have available z score but no data on the 3-gene classifier and vice versa. Number of cases with z score above 2 are depicted for each gene. In parentheses are percentages of patients with z scores above 2 for each gene of the amplicon and for each breast cancer sub-type.

Gene	ER+/HER2-/Proliferation High with z Score >2 (%)	ER+/HER2-/Proliferation Low with z Score >2 (%)	HER2+ with z Score >2 (%)	ER-/HER2- with z Score >2 (%)	Total Cases with z Score >2 (%)
Total cases with data available	150	63	22	18	
ERLIN2	64 (42.7%)	18 (28.6%)	2 (9.1%)	2 (11.1%)	93 (34.1%)
ZNF703	46 (30.7%)	5 (7.9%)	2 (9.1%)	1 (5.6%)	61 (22.3%)
PLPBP	58 (38.7%)	17 (27%)	1 (4.5%)	2 (11.1%)	85 (31.1%)
ADGRA2	1 (0.6%)	4 (6.3%)	0	3 (16.7%)	9 (3.2%)
BRF2	69 (46%)	17 (27%)	1 (4.5%)	3 (16.7%)	99 (36.2%)
RAB11FIP1	58 (38.7%)	13 (2.1%)	3 (13.6%)	1 (5.6%)	83 (30.4%)
GOT1L1	1 (0.7%)	1 (1.6%)	0	0	2 (0.7%)
ADRB3	3 (2.0%)	0	3 (13.6%)	0	7 (2.5%)
EIF4EBP1	41 (27.3%)	6 (9.5%)	5 (22.7%)	9 (50%)	69 (25.2%)
ASH2L	59 (39.3%)	17 (27%)	2 (9.1%)	4 (22.2%)	89 (32.6%)
STAR	4 (2.7%)	1 (1.6%)	0	2 (11.1%)	8 (2.9%)
LSM1	60 (40%)	17 (27%)	2 (9.1%)	5 (27.8%)	92 (33.6%)
BAG4	45 (30%)	8 (12.7%)	1 (4.5%)	3 (16.7%)	64 (23.4%)
DDHD2	49 (32.7%)	14 (22.2%)	3 (13.6%)	6 (33.3%)	79 (28.9%)
PLPP5	47 (31.7%)	14 (22.2%)	2 (9.1%)	1 (5.6%)	69 (25.2%)
NSD3	61 (40.7%)	14 (22.2%)	3 (13.6%)	3 (16.7%)	89 (32.6%)
LETM2	30 (20%)	2 (3.2%)	0	0	38 (13.9%)
FGFR1	33 (22%)	12 (19.4%)	2 (9.1%)	1 (5.6%)	54 (19.8%)
TACC1	9 (6.0%)	5 (7.9%)	3 (13.6%)	1 (5.6%)	19 (6.9%)

**Table 6:** Number of putative promoter sites for various transcription factors. Promoter sequences are considered from aminoacid -1000 upstream the Transcription Start Site (TSS) to aminoacid 100 after TSS (*p* value < 0.001). Promoters are from the Eukaryotic Promoter Database (EPD, www.epd.epfl.ch). Transcription factor target sequences are from the JASPAR database (www.jaspar.genereg.net). Each number refers to the number of putative binding sites for each transcription factor in a given promoter, so that genes with more than one promoter (up to 4 for FGFR1) listed in EPD have more than one numbers listed for each transcription factor.

	ESR1	ESR2	E2F1	Nfe2l2	GATA3	FOXA1
ERLIN2	6, 6	2, 3	2, 2	1, 1	1, 1	1, 1
ZNF703	5	1	4	0	0	0
PLPBP	5	3	2	3	0	1
ADGRA2	4, 8	6, 2	4, 6	1, 0	0, 0	0, 0
BRF2	3	2	4	4	0	1
RAB11FIP1	4, 2	3, 5	2, 2	4, 3	4, 0	1, 0
GOT1L1	3	6	0	5	2	2
ADRB3	4	2	2	3	0	1
EIF4EBP1	4, 1	2, 2	2, 2	1, 1	0, 2	1, 1
ASH2L	0, 4	0, 0	3, 3	2, 3	2, 1	2, 0
STAR	6, 6, 4	2, 3, 4	1, 3, 0	3, 3, 2	1, 0, 0	3, 5, 1
LSM1	5, 4	4, 5	4, 4	3, 2	0, 0	0, 0
BAG4	3, 2	7, 4	4, 4	1, 0	1, 1	0, 2
PLPP5	2	4	2	1	0	2
NSD3	10, 6	5, 4	6, 7	3, 4	1, 0	0, 0
LETM2	2, 2	2, 1	8, 9	3, 4	2, 2	2, 2
FGFR1	4, 8, 3, 8	2, 2, 2, 2	4, 10, 2, 10	2, 1, 3, 1	0, 0, 0, 0	1, 0, 1, 0
TACC1	3, 5, 4	4, 1, 3	3, 10, 2	2, 1, 4	1, 0, 1	3, 1, 0

**Table 7:** Protein expression by Immunohistochemistry (IHC) from the human protein atlas. In IHC staining columns, intensity of staining has been grouped as none/low and medium/high. The second column shows the antibody commercial catalogue number, type and company.

Protein	Primary Antibody	IHC Staining (Number of Samples)	
		None-Low	Medium-High
ERLIN2	HPA002025 (r pAb) Sigma-Aldrich	0	11
	CAB014894 (r mAb) Origene	0	12
ZNF703	HPA023930 (r pAb) Sigma-Aldrich	9	2
	CAB068249 (m mAb) Sigma-Aldrich	1	11
PLPBP	HPA023646 (r pAb) Sigma-Aldrich	9	2
	HPA023733 (r pAb) Sigma-Aldrich	5	6
	CAB017033 (m mAb) Origene	5	5
ADGRA2	HPA012393 (r pAb) Sigma-Aldrich	11	0
BRF2	HPA023378 (r pAb) Sigma-Aldrich	0	11
	CAB019269 (r mAb) Origene	9	2
RAB11FIP1	HPA023904 (r pAb) Sigma-Aldrich	2	10
	HPA024010 (r pAb) Sigma-Aldrich	3	8
	HPA025960 (r pAb) Sigma-Aldrich	1	9
	CAB017037 (r mAb) Origene	0	12
GOT1L1	HPA028778 (r pAb) Sigma-Aldrich	0	12
ADRB3	HPA061969 (r pAb) Sigma-Aldrich	12	0
EIF4EBP1	HPA023501 (r pAb) Sigma-Aldrich	5	6
	CAB005032 (r mAb) Epitomics	0	12
	CAB005039 (r mAb) Epitomics	7	5
ASH2L	HPA042289 (r pAb) Sigma-Aldrich	1	10
STAR	HPA023644 (r pAb) Sigma-Aldrich	9	0
	HPA027318 (r pAb) Sigma-Aldrich	9	0
	CAB032598 (r pAb) Santa Cruz Biotechnology	9	0
LSM1	-		
BAG4	HPA018951 (r pAb) Sigma-Aldrich	0	11
	CAB013716 (r mAb) Origene	0	12
DDHD2	HPA023143 (r pAb) Sigma-Aldrich	10	1
	HPA023147 (r pAb) Sigma-Aldrich	8	2
	CAB015202 (r mAb) Origene	0	12
PLPP5	-		
NSD3	HPA005659 (r pAb) Sigma-Aldrich	4	8
	HPA018893 (r pAb) Sigma-Aldrich	0	11
	CAB013721 (r mAb) Origene	0	11
LETM2	HPA025032 (r pAb) Sigma-Aldrich	5	6
FGFR1	HPA056402 (r pAb) Sigma-Aldrich	12	0
	CAB033614 (m mAb) Santa Cruz Biotechnology	0	11
TACC1	HPA024702 (r pAb) Sigma-Aldrich	3	9
	CAB017041 (r mAb) Origene	6	6

r pAb: Rabbit polyclonal antibody, r mAb: Rabbit monoclonal antibody, m mAb: Mouse monoclonal antibody, SA: Sigma-Aldrich, O: Origene, E: Epitomics, SCB: Santa Cruz Biotechnology.

**Table 8:** Frequency of amplification of the 8p11.23 genes in metastatic breast cancer studies. The metastatic breast cancer project included three samples with deletions in all amplicon genes. Mutations were observed in zero to three cases in each amplicon gene. The INSERM metastatic breast cancer study included one to four patients with deletions and zero to three patients with mutations in genes of the amplicon.

Gene	Metastatic Breast Cancer Project	INSERM Study
Total	*n* = 180 (%)	*n* = 216 (%)
ERLIN2	29 (16.1%)	34 (15.7%)
ZNF703	37 (20.6%)	34 (15.7%)
PLPBP	28 (15.6%)	35 (16.2%)
ADGRA2	26 (14.4%)	33 (15.3%)
BRF2	25 (13.9%)	34 (15.7%)
RAB11FIP1	24 (13.3%)	34 (15.7%)
GOT1L1	22 (12.2%)	32 (14.8%)
ADRB3	22 (12.2%)	32 (14.8%)
EIF4EBP1	23 (12.8%)	28 (13%)
ASH2L	21 (11.7%)	28 (13%)
STAR	19 (10.6%)	28 (13%)
LSM1	19 (10.6%)	28 (13%)
BAG4	18 (10%)	28 (13%)
DDHD2	18 (10%)	27 (12.5%)
PLPP5	19 (10.6%)	27 (12.5%)
NSD3	18 (10%)	27 (12.5%)
LETM2	21 (11.7%)	29 (13.4%)
FGFR1	15 (8.3%)	29 (13.4%)
TACC1	12 (6.7%)	23 (10.6%)
